# 

**Published:** 1923-09

**Authors:** 


### HOSPITAL and HEALTH REVIEW

Established 1886 No. 1866. Vol. LXXII.

New Series. Vol. II No. 24 September, 1923. Price Sixpence.

CONTENTS.

| | PAGE |
|---|---|
| Talking Health and Acting Health . . . . | 321 |
| The Pay of Medical Officers . . . . | 322 |
| Notes and Comments . . . . . . . | 323 |
| Payment of Hospital Staffs—Ice as a Luxury—Food Poisoning and Food Infection—The Gloucester Outbreak—Hand-In-Hand—Occupational Therapy in Hospital Practice—Fewer Flies—Consolidation of Health and Housing Laws—Failure to Notify Tuberculosis—Black Smoke and Grey—Re-Assuring the Assured—The Human Carriage—Heliotherapy—The Future of Bethlem Hospital—Dental Caries. | |
| The Head-to-Foot Way . . . . . . | 327 |
| By Sir Alfred Yarrow, F.R.S. | |
| Metropolitan Asylums Board Report . . . | 328 |
| The Medical Officer: His Professional Education and Training . . . . . . | 329 |
| First Aid for the Cinema "Artist" . . . | 330 |
| The Army Medical Service . . . . . | 331 |
| By a Man in Retirement. | |
| An East Coast Convalescent Home . . . . | 332 |
| The Dreyer Tuberculosis Vaccine . . . . | 332 |
| September and the Panel Doctor . . . | 333 |
| Internationalising Health . . . . . | 334 |
| The Public Health: Interviews with Local Authorities—Leicester . . . . | 335 |
| How a Hospital Saves Premature Infants . | 337 |
| Health Insurance Notes . . . . . . | 338 |
| Hospital Progress and Finance . . . . | 339 |
| The Nation's Health . . . . . . | 341 |
| Correspondence . . . . . . . | 342 |
| Reviews of Books . . . . . . . | 343 |
| The Medical Curriculum . . . . . | 345 |
| Echoes of the Month . . . . . . | 351 |
| Hospital and Health News . . . . . | 352 |
| Public Health in Parliament . . . . | 354 |
| Recent Appointments . . . . . . | 354 |

